# Determining Insulin Resistance Cutoffs in Mexican Adults: Percentile Distribution vs. Receiver Operating Characteristic Curve Analysis

**DOI:** 10.7759/cureus.79775

**Published:** 2025-02-27

**Authors:** Maria Fernanda Decaro-Fragoso, Teresa Estrada-Garcia, Catalina Lopez-Saucedo, Cesar Ivan Elizalde-Barrera

**Affiliations:** 1 Department of Molecular Biomedicine, Center for Research and Advanced Studies of the National Polytechnic Institute, Mexico City, MEX; 2 Department of Internal Medicine, General Hospital of Zone No. 30, Mexican Social Security Institute, Mexico City, MEX

**Keywords:** cutoff values, insulin resistance, metabolic syndrome, percentile analysis, population-specific reference

## Abstract

Introduction

Insulin resistance (IR) plays a key role in the development of metabolic syndrome (MetS), type 2 diabetes, and cardiovascular disease. The Homeostasis Model Assessment of Insulin Resistance (HOMA-IR) is widely used to estimate IR, but there is no consensus on the optimal cutoff values for identifying individuals at risk. This study aims to compare two methodologies, percentile distributions and receiver operating characteristic (ROC) curve analysis, for determining optimal HOMA-IR cutoff values in a population from Mexico City.

Methods

This cross-sectional study included 765 adults recruited from a hospital outpatient clinic in Mexico City. Participants were divided into two groups: a reference group of individuals with healthy weight and fasting plasma glucose and a MetS group of overweight or obese individuals classified based on the presence or absence of MetS. HOMA-IR values were analyzed using the 75th percentile in the reference group and ROC curve analysis in the MetS group. Optimal cutoffs were determined using the Youden index.

Results

We include a total of 765 patients, 218 subjects in the reference group and 547 for the ROC curve analysis. HOMA-IR percentiles 75th and 90th were 2.72 and 3.71, respectively. ROC curve analysis yielded higher cutoff values for MetS diagnosis than the percentile-based method. The percentile-based approach allowed for earlier identification of individuals at risk, including those without clinical manifestations of MetS.

Conclusions

This study highlights the variability in HOMA-IR cutoff values across methodologies and emphasizes the importance of population-specific reference values. A percentile-based approach proves effective for early detection of IR, facilitating preventive interventions during the preclinical stage. These findings support using percentile-based cutoffs as a practical tool for improving risk assessment and guiding clinical decision-making.

## Introduction

Insulin resistance (IR) is a complex pathological condition characterized by a reduced physiological response of peripheral tissues to normal insulin levels. IR plays a crucial role in the pathogenesis of metabolic diseases such as type 2 diabetes (T2D) and metabolic syndrome (MetS) [1,2]. Evaluating IR is valuable in clinical practice as it helps identify high-risk patients who may benefit from prevention strategies [3]. The euglycemic-hyperinsulinemic clamp method is considered the gold standard for accurately assessing IR in clinical and research settings [4] but its use is limited in clinical practice due to the time and cost involved. The Homeostasis Model Assessment of Insulin Resistance (HOMA-IR), described by Matthews et al. in 1985 [5], is a simple and widely used method for estimating IR in large epidemiological studies and clinical settings [6,7].

A reference value is essential for the clinical interpretation of HOMA-IR. However, despite its widespread use, there is no consensus on optimal cutoff values. Several studies have attempted to define cutoff values for diagnosing IR in various populations, reporting considerable variability in thresholds based on ethnicity, methods of estimation, and metabolic conditions of the populations studied [8-10].

Two common methods for determining cutoff values include using percentile distributions of HOMA-IR (e.g., the 75th or 90th percentiles) and receiver operating characteristic (ROC) curve analysis to discriminate MetS. However, the optimal method remains a subject of debate. Both methods have advantages and disadvantages: ROC curve analysis could identify individuals with the IR phenotype, although IR may not be at the core of the cluster of metabolic abnormalities that characterizes MetS. On the other hand, HOMA-IR cut-off obtained from a percentile approach may be limited to identify individuals at risk of metabolic complications.

Furthermore, different definitions of MetS, which use varying combinations of diagnostic criteria, have been proposed [11]. Whether these definitions are related to IR has not been sufficiently evaluated.

This study aims to compare HOMA-IR cut-off values determined using two approaches: (i) percentile distributions in a reference population and (ii) ROC curve analysis. In the second approach, the cutoff values were used to identify individuals with MetS based on two definitions: the 2009 harmonized worldwide consensus statement by the American Heart Association/National Heart, Lung, and Blood Institute (AHA/NHLBI) [12] and the World Health Organization (WHO) [13] criteria in an adult population from Mexico City.

## Materials and methods

This cross-sectional study was conducted through a collaboration between General Hospital No. 30 Iztacalco and the Center for Research and Advanced Studies of the National Polytechnic Institute (CINVESTAV-IPN). The study protocol was approved by Local Health Research Committee 3703 Family Medicine Unit Number 21 (approval number: R-2023-3703-047) and adhered to the Declaration of Helsinki and local ethical standards. Informed consent was obtained from all participants before enrollment.

Study inclusion criteria were patients with age ≥ 18, either sex, BMI> 18.5 kg/m^2^, who agreed to participate in the study. We used a simple random sampling to select participants from the outpatient clinic of General Hospital No. 30 Iztacalco, part of the Mexican Social Security Institute, in Mexico City, from May 2023 to June 2024. Individuals with a history of recent acute illness or known cardiovascular, hepatic, renal, or endocrine disease were excluded. Pregnant or breastfeeding women, individuals receiving treatment for diabetes or dyslipidemia, and smokers were also excluded.

After recruitment, participants were allocated into one of two groups: (i) to determine percentile-based cutoff values, a population of individuals with healthy weight according to WHO criteria (body mass index <25 kg/m²) and normal fasting plasma glucose (FPG) levels according to American Diabetes Association (ADA) criteria: FPG <100 mg/dL, was selected (reference group) and (ii) for the ROC curve analysis approach, overweight or obese individuals (body mass index 25-30 kg/m² or >30 kg/m² respectively, WHO criteria) were categorized based on the presence or absence of metabolic syndrome according two definitions: AHA/NHLBI and WHO (MetS group).

Clinical and laboratory measurements

Body weight and height were measured using a standard protocol with participants standing, without shoes, and wearing light clothing. Waist circumference was measured in the standing position between the iliac crest and rib cage at the end of expiration. Body mass index was calculated as weight (kg) divided by the square of height (m²).

Blood samples were collected in the morning after at least eight hours of overnight fasting. FPG, high-density lipoprotein cholesterol (HDL-C), and triglycerides (TG) were analyzed using an automated biochemistry analyzer (Hitachi Inc., Tokyo, Japan) with commercial reagents at the clinical laboratory of General Hospital Iztacalco. Fasting insulin levels were measured using an enzyme-linked immunosorbent assay (Calbiotech, CA, USA, catalog number IN374S) in the research laboratory of the Department of Molecular Biomedicine at CINVESTAV-IPN. The HOMA-IR was calculated using the following equation:

$\text{HOMA-IR} = \frac{\text{Fasting Insulin (IU/mL)} \times \text{FPG (mg/dL)}}{405}$

Definitions

MetS was defined using the following criteria: central obesity (waist circumference ≥90 cm for men and ≥80 cm for women, based on cut-off points for the Mexican population), TG ≥150 mg/dL, HDL-C <40 mg/dL (men) or <50 mg/dL (women), systolic blood pressure ≥130 mmHg or diastolic blood pressure ≥85 mmHg, and impaired fasting glucose (IFG; FPG ≥100 mg/dL and <126 mg/dL). IFG was defined according to the ADA diagnostic criteria [14]. Under the AHA/NHLBI criteria, MetS was diagnosed if three or more criteria were met. According to the WHO definition, MetS was diagnosed if IFG was present along with two or more components.

Statistical analysis

Data were analyzed using IBM SPSS Statistics for Windows, Version 26 (Released 2019; IBM Corp., Armonk, New York, United States). The normality of quantitative data distribution was assessed using the Kolmogorov-Smirnov test. Normally distributed data are presented as means ± standard deviation, while non-normally distributed data are presented as medians and interquartile ranges. Categorical data are expressed as prevalence (%).

For the first approach (reference group), the distribution of HOMA-IR values was tabulated for the 25th, 50th, 75th, and 90th percentiles using parametric procedures. The percentile 75th value in the reference group was considered the optimal cut-off. For the second approach (MetS group), ROC curves were constructed to evaluate the predictive value of HOMA-IR for MetS in both definitions used, AHA/NHLBI and WHO. The area under the curve (AUC) with corresponding 95% confidence intervals (CI) was calculated. Optimal cutoff values were determined using the Youden index, defined as sensitivity + specificity − 1, to identify the cutoff point with the highest combined sensitivity and specificity. A p-value < 0.05 was considered statistically significant.

## Results

A total of 765 participants were included, divided into 218 in the reference group and 547 in the MetS group. The clinical and metabolic characteristics of both groups are summarized in Table 1. The MetS group included 319 women and 228 men, while the reference group included 140 women and 78 men. The median age was 36.2 years for the reference group and 46.7 years for the MetS group.

**Table 1:** General characteristics of the study population MetS, metabolic syndrome; BMI, body mass index; TG, triglycerides; HDL-C, high-density lipoprotein cholesterol; IQR, interquartile range; HOMA-IR, homeostasis model assessment of insulin resistance

Variable	All	Reference Group	MetS Group
N	765	218	547
Women (n, %)	459 (60%)	140 (64%)	319 (58.4%)
Men (n, %)	306 (40%)	78 (36%)	228 (41.6%)
Median age, years (±SD)	41.6 (±13.4)	36.2 (±13.1)	46.7 (±12.16)
Mean weight, kg (±SD)	74.28 (±14.6)	60.1 (±8.19)	79.4 (±11.6)
Mean height, m (±SD)	1.62 (±10.5)	1.62 (±8.71)	1.6 (±12.8)
Mean BMI, kg/m^2^ (±SD)	28.12 (±4.7)	22.81 (±1.75)	30.7 (±3.81)
Median glucose, mg/dL (IQR)	94.1 (86.75-104.75)	88.15 (81-95.2)	100.9 (±15.1)
Median HDL-C, mg/dL (IQR)	47.0 (40.0-55.0)	51.7 (44.0-59.07)	46.8 (±11.13)
Median TG, mg/dL (IQR)	133.0 (92.0-177.0)	95 (69.4-142.5)	153 (113-206.7)
Median insulin, IU/mL (IQR)	11.95 (6.43-18.5)	7.57 (4.0-12.6)	13.7 (8.9-19.3)
Median HOMA-IR (IQR)	2.73 (1.46-4.46)	1.27 (0.64-2.3)	3.2 (2.03-5.2)

HOMA-IR and fasting insulin percentiles for the whole study population and reference group are shown in Table 2. For the reference group, the 75th and 90th percentiles of HOMA-IR were 2.72 and 3.71, respectively. 

**Table 2:** HOMA-IR percentiles MetS, metabolic syndrome; HOMA-IR, homeostasis model assessment of insulin resistance.

Percentiles	All (n = 765)		Reference group (Percentile methodology n = 218)	
	Insulin	HOMA-IR	Insulin	HOMA-IR
P25th	6.43	1.46	3.9	0.82
P50th	11.95	2.73	7.57	1.56
P75th	18.5	4.46	12.64	2.72
P90th	27.07	6.43	17.7	3.71

Table 3 summarizes the results of the ROC analysis for the MetS group, including AUC, 95% CI, sensitivity, specificity, and Youden index values. The optimal HOMA-IR cutoff values for identifying MetS were 4.21 (sensitivity 54.8%, specificity 73.8%, AHA/NHLBI definition) and 4.23 (sensitivity 58.9%, specificity 71.7%, WHO definition). Using these cutoffs, 59% of participants meeting the WHO criteria for MetS and 55% of participants meeting the AHA/NHLBI criteria for MetS, were classified as having IR. 

**Table 3:** ROC curve analysis ROC, receiver operating characteristic; AUC, area under the curve; 95% CI, 95% confidence interval; MetS, metabolic syndrome; AHA/NHLBI, American Heart Association/National Heart, Lung, and Blood Institute; WHO, World Health Organization. ^a^Receiver operating characteristic curve analysis

Group	AUC	95% CI	Cutoff	Sensitivity (%)	Specificity (%)	Youden Index	P-Value^a^
MetS (AHA/NHLIB)	0.653	0.599-0.707	4.21	54.8	73.8	0.286	0.0001
MetS (WHO)	0.678	0.623-0.732	4.23	58.9	71.7	0.306	<0.0001

## Discussion

This study documented variations in the optimal cutoff values for the HOMA-IR based on the methodology used. Two common approaches for determining HOMA-IR cutoffs include clinical manifestations of IR (e.g., MetS) and percentile distributions (typically the 75th or 90th percentiles) in healthy reference populations. In our study, HOMA-IR cut-off values ranged from 2.7 (75th percentile) to 4.2 (to discriminate MetS).

Previous studies have reported discrepancies in HOMA-IR cutoffs based on percentile methodology across different populations. Lower cutoff values have been reported in Chinese (75th = 1.44; 90th = 2.03) [15], Japanese (90th = 1.7) [10], Portuguese (90th = 2.33) [16], Slovak rural (75th = 2.29) [17], and Iranian populations (75th = 1.6; 90th = 2.3) [9]. Our percentile-based cutoffs are more similar to intermediate values reported in Latin American populations, such as Chileans (75th = 2.57) [18], Argentines (75th = 2.64) [19], Brazilians (90th = 2.71) [8], and Spaniards (75th = 2.6; 90th = 3.8) [20,21]. Higher cutoff values have been reported in Koreans (75th = 3.04) [22] and in populations from southwestern France (75th = 3.8) [23].

Similar variations have been observed in HOMA-IR cutoffs for diagnosing MetS, with different MetS definitions contributing to this variability. For example, in Japanese populations, the cut-off was 1.7 [24], while in Portuguese populations, the cut-off was 2.41, using the AHA/NHLBI definition [16]. Higher cutoffs have been reported in young Polish individuals (4.22; International Diabetes Federation (IDF) 2009 definition) [25]. Among Korean subjects, cut-offs ranged from 2.34 (Adult Treatment Panel III (ATP III) definition) [22] to 1.22-1.28 (for men and women, respectively, using AHA/NHLBI criteria) [26].

Two studies compared IDF and ATP III criteria. Esteghamati et al. reported similar cut-offs for ATP III (1.95) and IDF (1.85) definitions in an Iranian population [9]. Lee et al. reported identical values for men and women using AHA/NHLBI and IDF criteria [26].

Potential mechanisms that could explain ethnic differences in IR are not completely understood. Furthermore, few studies have been conducted to assess IR between individuals with different ethnic backgrounds. However, several factors have been examined as possible determinants, including racial differences in body fat distribution, reduced hepatic insulin sensitivity, and plasma levels of adiponectin [27-30].

Determining specific cutoff values is important for enhancing the clinical utility of the HOMA-IR method and identifying patients with increased metabolic risk. However, there is no consensus on the optimal methodology, leading to a broad range of reported values. These differences suggest that IR varies among populations due to ethnicity and race, which influence susceptibility to IR [20,31]. Population-specific reference values are therefore necessary for accurate risk assessment and for planning preventive strategies targeting public health issues such as obesity, T2D, and cardiovascular disease.

MetS is characterized by multiple metabolic abnormalities that place individuals at high risk for diabetes and atherosclerosis [32]. Although the development of MetS is not fully understood, IR is proposed as the underlying mechanism driving these abnormalities [2]. However, whether MetS components share a common pathogenic process remains debated, and its pathophysiology likely involves complex mechanisms that require further investigation [33].

In our study, we found differences in the HOMA-IR cut-off values according to the methodology used, with higher HOMA-IR cut-off values observed in the curve ROC approach compared with the percentile-based approach. This difference may reflect a preclinical period of IR, during which FPG levels are within the reference range and MetS features are absent. Depending on individual susceptibility and the severity of IR, metabolic abnormalities develop over time. Screening for IR during this asymptomatic period could improve risk assessment and preventive management.

Patients with diabetes were excluded from our study in order to avoid potential bias due to the influence of treatment on IR assessment, although this selection criterion may limit the applicability of the results in patients with diabetes. Additionally, we acknowledge the limitations of the study, our sample could not be representative of the general adult population in Mexico, and the cross-sectional design prevents making causal inferences and conclusions about the predictive accuracy of HOMA-IR cutoffs for T2D or cardiovascular disease. Prospective, population-based studies are needed to address this issue.

## Conclusions

This study compared two methodologies, percentile distributions and ROC curve analysis, to determine HOMA-IR cutoff values and identify individuals at risk for MetS. We found a higher cutoff value in the ROC curve analysis compared with the percentile approach, suggesting more severe IR in patients with MetS as a clinical manifestation of IR. Therefore, our findings indicate that the percentile-based approach is more effective for early detection of IR, enabling the identification of individuals during the preclinical stage when preventive interventions can be most beneficial. By addressing the variability in HOMA-IR cutoffs across populations, this study highlights the need for population-specific reference values tailored to factors such as ethnicity and metabolic characteristics which could improve clinical decision-making and risk assessment. The percentile-based method offers a practical and cost-effective tool for improving risk assessment, facilitating early intervention, and potentially reducing the long-term burden of metabolic and cardiovascular diseases. These findings provide clinicians with a standardized framework to guide preventive care and enhance patient outcomes.
